# Azithromycin in the Successful Management of COVID-19: A Family Physician’s Perspective

**DOI:** 10.7759/cureus.14574

**Published:** 2021-04-20

**Authors:** Leonid Tafler, Anastasia Danilevsky, Divya Seth

**Affiliations:** 1 Primary Care, Touro College of Osteopathic Medicine, New York, USA; 2 Medicine, St. George’s University, St. George, GRD; 3 Family Medicine, Touro College of Osteopathic Medicine, New York, USA

**Keywords:** covid-19 outbreak, azithromycin, critical care, epidemiology, hospitalization, sars-cov-2, antiviral, supportive therapy, ambulatory treatment

## Abstract

The novel severe acute respiratory syndrome coronavirus 2 (SARS-COV-2), causing coronavirus disease-19 (COVID-19), has been responsible for approximately 75 million cases and 1.6 million deaths globally as of December 22, 2020. Currently, no treatment modalities or management options have been recommended by the National Institutes of Health (NIH) prior to patient hospitalization and supplemental oxygen requirement. This poses a unique challenge for outpatient primary care physicians, who are often tasked with initial care of patients early on in their disease course. During the pandemic, our family practice provided medical care to approximately 2,000 families located in the surrounding Brooklyn community. With only telemedicine at our disposal, our clinic was tasked with treating patients presenting remotely who may or may not have had COVID-19 - a large clinical diagnosis was made given the absence of in-person testing. Often co-administered, Azithromycin was considered a supportive agent that may or may not have increased the benefit of hydroxychloroquine. However, Azithromycin may perform well on its own for various reasons as it has been shown to have antiviral activity against other RNA viruses, anti-inflammatory properties, and antiviral effects within bronchial epithelial cells. Azithromycin has also shown efficacy as an add-on treatment for reducing asthma exacerbations - pertinent to the pro-inflammatory pulmonary conditions in COVID-19 progression - and may even prevent or treat bacterial co-infection in patients with SARS-COV-2.

In order to investigate the association between Azithromycin and the COVID-19 disease process, our clinical study retrospectively identified patients who were prescribed Azithromycin (500 mg on day one + 250 mg on days two to five) during the peak months of the COVID-19 pandemic in New York City from March 2020 through May 2020. All patients prescribed Azithromycin with suspicion of COVID-19 infection were interviewed via telephone regarding their constellation of symptoms, compliance with the prescribed antibiotic for the intended course, symptom duration prior to and following antibiotic course initiation, as well as any further complications of their illness, if present. Ultimately, the majority of the patients who were interviewed over the phone concluded that a full course of Azithromycin helped improve their symptoms during their infection with COVID-19. Outcomes and complications in patients treated with Azithromycin were noteworthy in that there were no reports of pulmonary complications or deterioration of pulmonary function after treatment (e.g., no shortness of breath, wheezing, dyspnea, etc.), although some patients did experience residual coughing and nasal discharge post-treatment. We believe further study of this treatment in the setting of experimental, randomized controlled trials may reveal the benefits of Azithromycin in terms of reducing infection severity, length, and limiting the incidence of complications in patients with COVID-19.

## Introduction

The novel severe acute respiratory syndrome coronavirus 2 (SARS-COV-2), causing coronavirus disease-19 (COVID-19), has been responsible for approximately 75 million cases and 1.6 million deaths globally as of December 22, 2020 [1]. Antecedent to patient hospitalization for COVID-19, no treatment modalities are currently recommended by the National Institutes of Health (NIH) for outpatient use [2]. This poses a unique challenge for outpatient primary care physicians, who are often tasked with initial care of patients early on in their disease course [3]. During the early stages of the pandemic in the United States, “stay-at-home” orders and social distancing requirements changed the nature of initial patient care from in-person to virtual telehealth visits [4]. Duke University School of Medicine reported an increase in their share of telehealth visits from <1% to 70% of visits in four weeks [4]. During this time, family physicians have been advised to screen their patients virtually for COVID-19 “red flags” (symptoms indicating moderate to severe disease), provide testing recommendations, counsel their patients regarding isolation precautions if disease is suspected, and provide supportive management [5,6]. During the pandemic, our family practice provided medical care to approximately 2,000 families located in the surrounding Brooklyn community. With only telemedicine at our disposal, our clinic was tasked with treating patients presenting remotely who may or may not have had COVID-19 - a large clinical diagnosis was made, given the absence of in-person testing. However, no treatments have been recommended for COVID-19 outpatient use nor for the early stages of the disease, leaving family practices like ours puzzled by how best to manage patients in the midst of a pandemic [5].

It has been proposed that viral loads in COVID-19 patients are highest at the time of symptom onset and decline thereafter; thus, in the event that COVID-19 is suspected at first contact with a primary care physician, it may be beneficial to explore pharmacologic therapy for early management of COVID-19 [6,7]. Several agents are currently being studied for this purpose. Among them, hydroxychloroquine was proposed to have properties that could aid COVID-19 treatment: interfering with viral replication, fever suppression, and immunomodulatory effects [8]. However, subsequent studies have revealed that there is not enough evidence for or against this agent in the treatment of COVID-19 [5]. Other treatment modalities being studied for early/outpatient use include various antiviral drugs, monoclonal antibodies, systemic corticosteroids, and some antibiotics (namely Azithromycin) [6,9]. Of these therapies, Azithromycin has been the treatment most commonly prescribed for COVID-19 in the outpatient setting and is associated with a well-tolerated adverse effect profile [10]. Often co-administered, Azithromycin was considered a supportive agent that may or may not have modulated the effects of hydroxychloroquine [10]. Ultimately, subsequent studies revealed that there is not enough evidence for or against the use of hydroxychloroquine in COVID-19; however, Azithromycin may perform well on its own for various reasons. It has been shown to have antiviral activity against other RNA viruses, anti-inflammatory properties, and antiviral effects within bronchial epithelial cells [9,11-13]. Azithromycin has also shown efficacy as an add-on treatment for reducing asthma exacerbations - pertinent to the pro-inflammatory pulmonary conditions in COVID-19 progression - and may even prevent or treat bacterial co-infection in patients with SARS-COV-2 [9,14].

In this observational study, we present the outcomes of patients with strong clinical suspicion of COVID-19 who presented to our family practice remotely for care. With no better treatment option available, patients were treated with Azithromycin, in addition to being given all recommended guidelines for self-isolation and testing, with strongly positive clinical responses and few complications.

## Materials and methods

In order to investigate the association between Azithromycin and the COVID-19 disease process, our clinical study identified patients who were prescribed Azithromycin (500 mg on day one + 250 mg on days two to five) during the peak months of the COVID-19 pandemic in New York City from March 2020 through May 2020. Patient information was collected through NextGen Office, the electronic medical record (EMR) being used by our clinic. Inclusion criteria for patients comprised an electronic medical record (EMR) record of Azithromycin prescription between March 23, 2020 and May 31, 2020 (n = 39). Once patients who fit the inclusion criteria were compiled, additional information was gathered, including the date Azithromycin was prescribed, patient history of exposure to COVID-19, history of COVID-19 antibody test, history of COVID-19 antigen test, and history of COVID-19 symptoms (e.g., shortness of breath, cough, mild fever, loss of taste/smell +/- abdominal complaints). Once patient information was compiled, patients were contacted to follow up on their symptoms. All patients prescribed Azithromycin with clinical suspicion of COVID-19 infection were interviewed via telephone regarding their constellation of symptoms, compliance with the prescribed antibiotic for the intended course, symptom duration prior to and following antibiotic course initiation, as well as any further complications of their illness, if present. Patients who were included in the study were not prescribed any other medication alongside Azithromycin, other than non-steroidal anti-inflammatory drugs (NSAIDs) or acetaminophen as supportive therapies. During the initial encounter, all patients were advised to self-quarantine at home under suspicion of being contagious with COVID-19.

## Results

**Figure 1:**
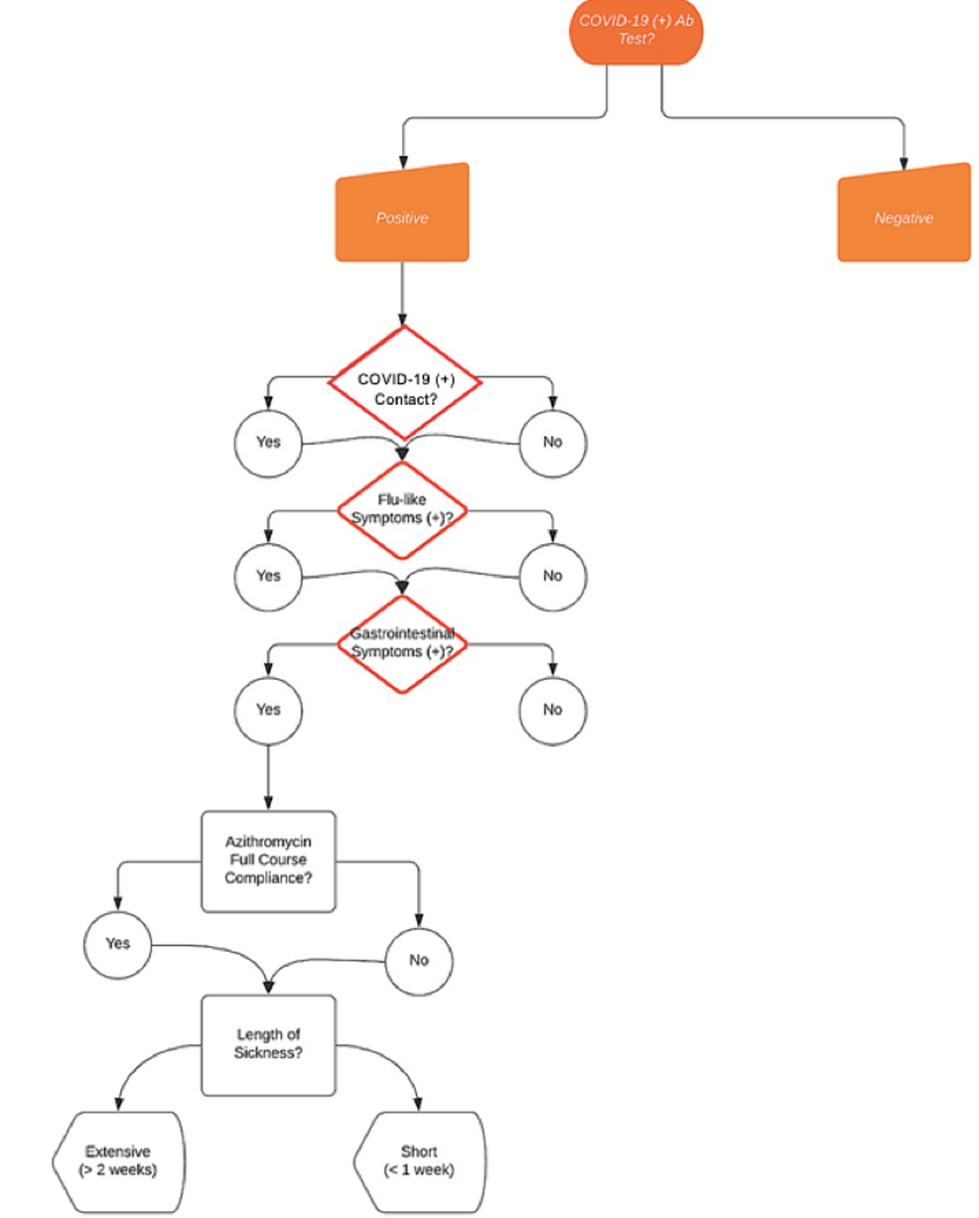
Preset questions flowchart

Of the 39 patients who were prescribed Azithromycin, 18 had also tested positive for COVID-19 antibodies at a follow-up visit. We deemed these patients likely to be COVID-19 positive at their initial telehealth visit based on their clinical presentation and their subsequent antibody results, and grouped them together as Group A. Each of these patients were contacted individually and interviewed over the phone. Patients were asked a number of preset questions (Figure 1), and their responses were recorded as shown in Table 1. Of the patients in Group A, 12 stated that the Azithromycin course decreased the intensity of their symptoms within one week, four stated that their symptoms persisted for exactly one week, and two patients reported their symptoms persisted for more than one week. Sixteen patients (n = 16) within Group A presented to their telemedicine appointment at the initiation of their symptoms, while two patients (n = 2) (#15 and #39) presented after symptoms had worsened.

**Table 1:** Patient response record GI, Gastrointestinal.

Patient #	Contact with COVID-19 (+)?	Flu-like symptoms?	GI symptoms?	Azithromycin compliance?	Length of sickness post-treatment?	Complications?
1	No	Yes	No	Yes	2 days	Abdominal pain
2	No	Yes	No	No	1 day	None
3	Yes	Yes	Yes	Yes	5 days	Slight cough
4	No	Yes	Yes	Yes	Few days	None
5	Yes	No	Yes	Yes	> 1 week	None
6	No	Yes	No	Yes	Few days	None
7	Yes	Yes	Yes	Yes	4 days	Slight cough
8	No	No	Yes	Yes	1 week	None
9	No	Yes	No	Yes	Few days	None
10	No	No	No	No	None	None
11	No	Yes	No	Yes	Few days	Abdominal pain
12	Yes	Yes	No	Yes	1 week	Runny nose
13	No	Yes	Yes	Yes	Few days	None
14	No	No	No	Yes	1 day	None
15	Yes	Yes	Yes	Yes	Few days	Slight cough
16	No	Yes	Yes	Yes	5 days	None
17	Yes	Yes	No	Yes	< 1 week	None
18	Yes	Yes	Yes	Yes	4 days	Runny nose
19	Yes	No	Yes	Yes	Few days	Postnasal drip
20	No	Yes	No	Yes	2 days	None
21	No	Yes	No	Yes	3 days	Diarrhea
22	Yes	Yes	Yes	Yes	< 1 week	Slight cough
23	Yes	Yes	Yes	Yes	Few days	Slight cough
24	No	No	Yes	Yes	2 days	None
25	No	Yes	No	Yes	1 day	None
26	Yes	Yes	Yes	Yes	Few days	Runny nose
27	Yes	Yes	Yes	Yes	> 1 week	None
28	No	Yes	No	No	Few days	None
29	No	No	Yes	Yes	2 days	None
30	No	Yes	No	Yes	2 days	None
31	No	Yes	No	Yes	1 day	None
32	Yes	Yes	Yes	Yes	Few days	None
33	No	Yes	No	Yes	Few days	None
34	Yes	Yes	No	Yes	Few days	None
35	No	Yes	No	Yes	< 1 week	None
36	No	No	Yes	No	3 days	None
37	Yes	Yes	No	Yes	1 day	None
38	Yes	Yes	Yes	Yes	1 week	None
39	Yes	Yes	No	Yes	1 week	None

Patients who were prescribed Azithromycin during the peak of the pandemic with unknown COVID-19 antibody status were referred to as Group B. Some of the patients were able to provide us with further information of their COVID-19 antibody testing status, which was positive, however, unconfirmed by the clinic. The rest of the patients were asked regarding their symptoms during the peak months of pandemic and whether they were tested for antibodies or whether they believed that they were sick with COVID-19. Some of the patients were tested and results were negative, and some patients believed that they were infected with COVID-19 but were never tested during or after their sickness (Table 2).

**Table 2:** Azithromycin original table

Patient	Azithromycin Prescription Date	COVID Antibody Test Date	Time Between	COVID Antibody Test Results
1	3/25/2020	5/20/2020	56	Negative
2	3/23/2020	4/28/2020	36	Negative
3	3/24/2020	8/11/2020	140	Positive
4	4/2/2020	6/29/2020	88	Positive
5	3/24/2020	8/11/2020	140	Positive
6	3/27/2020	5/22/2020	56	Negative
7	3/25/2020	5/9/2020	45	Positive
8	3/25/2020	9/11/2020	170	Positive
9	3/25/2020	5/26/2020	62	Negative
10	3/30/2020	4/24/2020	25	Negative
11	3/25/2020	4/27/2020	33	Negative
12	4/1/2020	5/13/2020	42	Positive
13	3/26/2020	5/2/2020	37	Negative
14	3/27/2020	5/22/2020	56	Negative
15	4/2/2020	4/20/2020	18	Positive
16	3/30/2020	9/22/2020	176	Positive
17	3/31/2020	10/9/2020	192	Positive
18	4/1/2020	5/20/2020	49	Positive
19	4/2/2020	4/22/2020	40	Positive
20	4/3/2020	6/1/2020	20	Negative
21	4/6/2020	10/21/2020	59	Negative
22	4/14/2020	10/24/2020	198	Positive
23	4/6/2020	7/8/2020	193	Positive
24	4/6/2020	6/5/2020	93	Negative
25	4/8/2020	4/29/2020	60	Negative
26	4/8/2020	6/30/2020	21	Positive
27	4/8/2020	6/30/2020	83	Positive
28	5/13/2020	7/8/2020	56	Negative
29	4/9/2020	8/30/2020	82	Negative
30	4/10/2020	5/4/2020	24	Negative
31	4/13/2020	4/30/2020	17	Negative
32	4/15/2020	7/12/2020	88	Positive
33	4/20/2020	5/4/2020	14	Negative
34	4/22/2020	4/22/2020	0	Negative
35	5/4/2020	5/4/2020	0	Negative
36	5/1/2020	4/30/2020	-1	Negative
37	4/30/2020	4/30/2020	0	Negative
38	5/13/2020	4/22/2020	21	Positive
39	4/25/2020	6/27/2020	45	Positive

## Discussion

Interest in this observational research study originated from the difficulties our practice faced in treating patients with a high clinical suspicion of COVID-19 via telemedicine. During the peak of the pandemic, many patients sought care from their primary care physicians and presented remotely with new-onset COVID-like symptoms. However, two of our patients presented to our clinic after developing symptoms that had led to a visit to the Emergency Department. In the Emergency Department, these patients were tested for COVID-19 and were instructed to quarantine with supportive therapy and fever suppressants. These patients were then contacted by the Emergency Department within a few days with news of a positive COVID-19 test result and were instructed to continue self-quarantining at home for the next 14 days. During this time, quarantined patients were divided between those who recovered versus those whose condition worsened, requiring further hospitalization and, in some cases, ventilatory support.

One of the patients interviewed from Group A, Patient #15, presented with this particular pattern of disease course. Patient #15 originally presented to her local Emergency Department with COVID-like symptoms and was promptly tested for COVID-19. After being tested, the patient was advised to return home, self-isolate, and wait for the results. The patient stated that she did as she was advised, and after two days, she was contacted by the hospital and notified that she tested positive for COVID-19. She was mandated to stay home and self-quarantine for 14 days. The patient was advised to stay on bed rest during this time period and to maintain supportive care. During the quarantine period, the patient’s symptoms continued to worsen until the patient decided to reach out to her primary care physician at our clinic for a telemedicine appointment. At the time of her visit, the patient’s condition had deteriorated: She described severe shortness of breath, difficulty taking deep breaths, and a severe cough. Fearing secondary opportunistic bacterial infection and further deterioration of the patient’s condition, the patient was prescribed Azithromycin. When interviewed, the patient stated that she felt better within days of taking the antibiotic. The patient was able to complete the full course of antibiotics and was under the impression that if not for the antibiotic, she would have been ventilated within a matter of days.

Encouraged, our clinic’s treating physician continued to prescribe Azithromycin, weighing its benefits (e.g., treating opportunistic bacterial infections, reducing chronic respiratory tract disease exacerbations, and its potential antiviral and immunologic properties) against its drawbacks (e.g., the patient’s potential for antibiotic resistance, Azithromycin’s adverse effect profile). Social distancing and supportive therapies aside, there were no other outpatient guidelines during the peak of the COVID-19 pandemic; thus, the encouraging history of Azithromycin use (as detailed previously) did not hold insignificant weight in treating our patients with no other recourse and worsening COVID-19 symptoms. Ultimately, the majority of the patients who were interviewed over the phone concluded that a full course of Azithromycin helped improve their symptoms during their infection with COVID-19. Outcomes and complications in patients treated with Azithromycin were noteworthy in that there were no reports of pulmonary complications or deterioration of pulmonary function after treatment (e.g., no shortness of breath, wheezing, dyspnea, etc.), although some patients did experience residual coughing and nasal discharge post-treatment.

While the authors do feel that, among our sample patient population, Azithromycin may have decreased the severity of COVID-19 symptoms and/or reduced the longevity of the disease process, this study does have several limitations to address. Our sample population does not have enough power to represent the entirety of New York City's COVID-19 patient population, as our clinic did not have the access to reach many numbers of patients during the pandemic. Further, this study does not demonstrate definitively that Azithromycin is an effective treatment for COVID-19 as the study is structured as an observational study and cannot determine causative relationships, only correlative ones. Finally, we cannot definitively claim that our patient sample did indeed have COVID-19 prior to being prescribed Azithromycin rather than afterward as we were unable to conduct COVID-19 tests on initial presentation due to pandemic restrictions. Our conclusions are thus based on strong clinical suspicion that patients presented with COVID-19 due to clinical presentation and COVID-19 antibody results upon follow-up.

## Conclusions

This observational study was conducted to demonstrate the efficacy of Azithromycin in treating COVID-19 from the outpatient perspective of a primary care provider. Especially when prescribed at the onset of patient symptoms, Azithromycin was well-tolerated, and the patients demonstrated improvement of symptoms within seven days. In many cases, Azithromycin use was even correlated with reversing the deterioration of patients who reported worsening of their symptoms in quarantine.

Due to the novel nature of this pandemic, our reliance on telemedicine, and the observational, retrospective structure of this study, we acknowledge that our options for patient randomization, control groups, and blinding were limited. However, the patients we treated with Azithromycin reported no adverse effects, and there were no cases of decompensation or further deterioration in any of our patients. Thus, we believe our observations may be of use in opening doors for further discussion and potential use of Azithromycin in the outpatient setting during symptom onset in patients with suspected COVID-19 infection. We believe further study of this treatment in the setting of experimental, randomized controlled trials may reveal the benefits of Azithromycin in terms of reducing infection severity, length, and limiting the incidence of complications in patients with COVID-19.
